# A nationwide pest risk analysis in the context of the ongoing Japanese beetle invasion in Continental Europe: The case of metropolitan France

**DOI:** 10.3389/finsc.2022.1079756

**Published:** 2022-12-12

**Authors:** Sylvain Poggi, Nicolas Desneux, Hervé Jactel, Christine Tayeh, François Verheggen

**Affiliations:** ^1^ IGEPP, Institut Agro, Univ Rennes, INRAE, Le Rheu, France; ^2^ University of Côte d’Azur, INRAE, CNRS, UMR ISA, Nice, France; ^3^ INRAE, University of Bordeaux, UMR Biogeco, Cestas, France; ^4^ Expertise and Biological Risks Unit (ERB), Plant Health Laboratory, French Agency for Food, Environmental and Occupational Health and Safety (ANSES), Angers, France; ^5^ TERRA, Gembloux Agro-Bio Tech, University of Liege, Gembloux, Belgium

**Keywords:** *Popillia japonica*, biological invasion, risk assessment, surveillance, monitoring, pest control, metropolitan France

## Abstract

The Japanese beetle, *Popillia japonica*, is native to Japan and became established in North America in the early twentieth century. The beetle was detected in Europe, first in Italy in 2014 and then in Switzerland in 2017. Metropolitan France is at the forefront of the Japanese beetle threat, due to its geographical proximity to the European populations established in the Piedmont, Lombardy and Ticino regions. An express pest risk analysis for metropolitan France was therefore conducted. The most likely pathways for entry include (i) natural dispersion, (ii) trades of plant products with adherent soil and (iii) hitchhiking behaviour, leading to a high probability of entry. The spread rate of *P. japonica* was also evaluated as high, resulting from natural spread as well as human activities. Given the absence of significant limiting factors, the potential impacts of *P. japonica* in France will likely be as important as in its current geographic distribution. Although several sources of uncertainty were highlighted throughout the evaluation, none of them has significant impact on the conclusions of the present express pest risk analysis. Measures to prevent entry, establishment and spread of *P. japonica* are recommended and include surveillance with pheromone traps and control *via* integrated pest management strategies. However, most efforts should be concentrated on eradication measures while *P. japonica* is still in the early stages of invasion.

## Introduction

The Japanese beetle, *Popillia japonica* Newman (Coleoptera, Scarabaeidae), is native to Japan (1). In the early twentieth century, it became established in North America, especially in the USA where it was initially introduced in the states bordering the Atlantic coast. Then, it quickly spread westwards (2) and became one of the worst invasive pests, inflicting severe damage to many cultivated and ornamental plants, trees, fruits, turfs and grasses. Its damage has been estimated to be $460 million per year in the USA (3).

Based on the severity of the economic, social and environmental impacts that *P. japonica* can cause in the European Union territory (4, 5), the European Commission classified *P. japonica* as a priority quarantine pest listed in Annex IIB of Regulation 2019/2072, subject to compulsory control and to a national sanitary emergency response plan, in accordance with European Regulation (EU) 2016/2031. Specific requirements are defined for imports of certain plants for planting because of their likelihood of harbouring *P. japonica*, depending on their origin ((EU) 2021/2285). Despite these efforts, the beetle was detected in Europe, first in Italy in 2014 (6, 7) and then in Switzerland in 2017 (8), two countries where it is now considered established. A few adults were also trapped in south-western Germany in 2021 and 2022 (9, 10), yet these sightings were considered as incursions without establishment.

The challenge for Europe is to counteract this invasion at a very early stage to significantly enhance the chances of successful eradication or containment. Because metropolitan France (i.e. mainland France and Corsica as well as nearby islands in the Atlantic Ocean, the English Channel and the Mediterranean Sea) is at the forefront of the *P. japonica* threat, the French Agency for Food, Environmental and Occupational Health & Safety (ANSES) was asked to carry out an express pest risk analysis (PRA thereafter; 11), based on the European and Mediterranean Plant Protection Organization (EPPO) express PRA scheme (12), in order to better and quickly prepare the State services for the implementation of measures in the case of a suspected and to implement control measures in the case of a confirmed outbreak. The presumed time horizon of the assessment is five years as suggested by EPPO (13). Following the recommended guidelines for performing an express PRA (12), we aimed at assessing the risks of entry, establishment, and spread of *P. japonica* in France as well as its potential impacts. Based on the overall risk, recommendations for surveillance and management measures in the event of an outbreak were formulated.

## Assessment of the phytosanitary risk

### Brief overview of the pest

**Figure 1:**
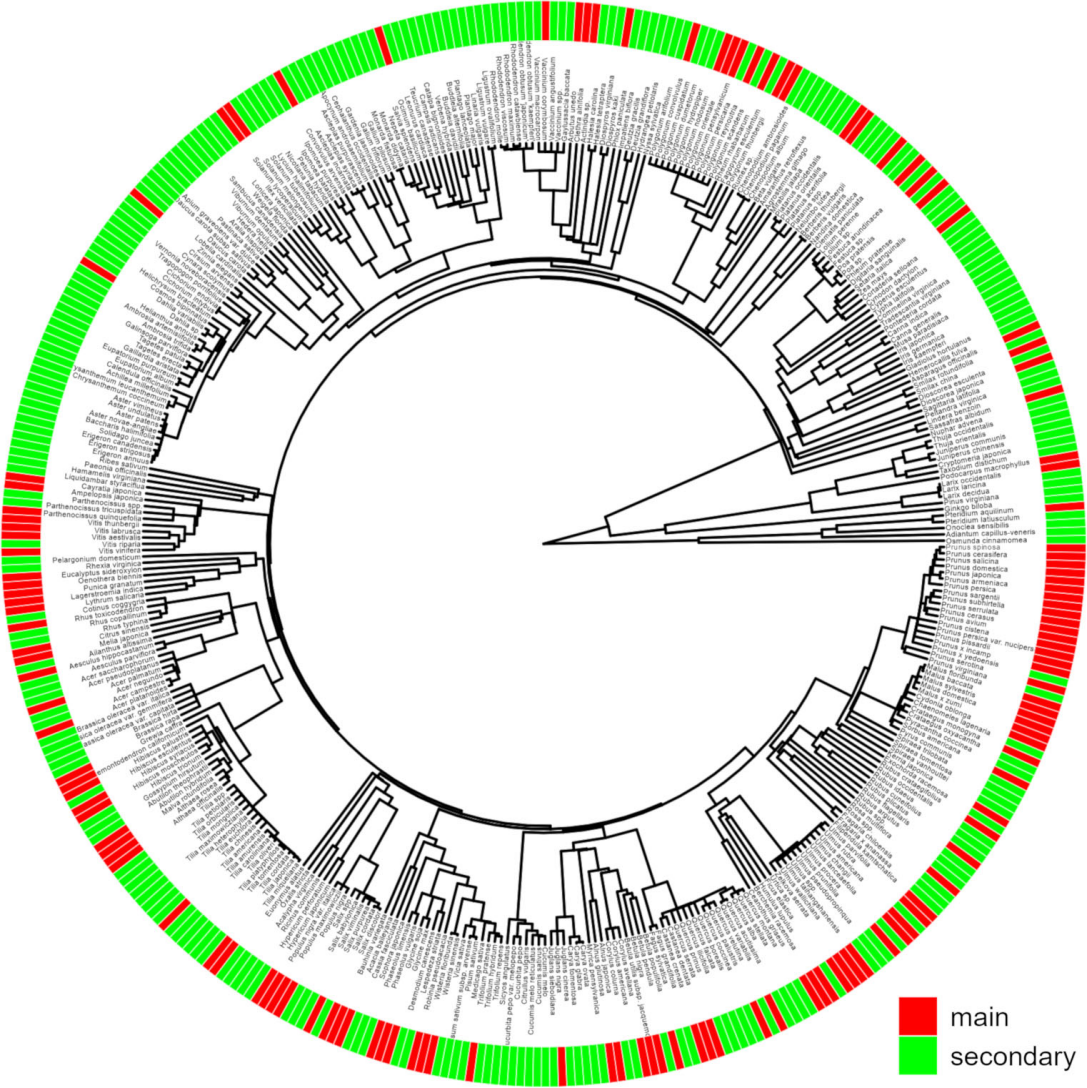
Phylogeny of the main and secondary host plants of *P. japonica* generated by the V.Phylomaker package (18). A total of 131 host plants, belonging to 39 families, are classified as “main” (17). This figure is also available as **Supplementary Material** to allow for magnification.

*Popillia japonica* is generally a univoltine species (as observed in Japan, Italy and Switzerland) but its development can spread over two years under colder climates (1, 14, 15). The beetle spends most of its life underground in immature forms (eggs, three larval stages and pupae) and a few months above ground as an adult. In Italy, adults are active from June to September with an activity peak in mid-July (16). *P. japonica* is a dietary generalist: larvae can feed on the rootlets of all host plants while adults preferentially feed on leaves but also on fruits and flowers. The symptoms caused by *P. japonica* adults are easily observed and consist of skeletonization, adults chewing the leaf tissue between the veins. An updated overview of the host plants of *P. japonica* (17) emphasises how polyphagous the beetle is (**Figure 1**): the adults feed on at least 401 host plants belonging to 92 botanical families including fruit trees (e.g. apple, plum), forest species (e.g. maple, poplar), field crops (e.g. corn, soybean) or vegetables (e.g. asparagus, beans), ornamental plants (e.g. roses), herbaceous species (species of the genus *Festuca*, *Lolium* and *Poa* used in lawns and turfs), wild species (e.g. clovers, brambles) and vines. Among this great diversity of host plants, Tayeh et al. (17) identify 131 species as “main” hosts, insofar as they favour the survival and reproduction of *P. japonica*. We focused on these main hosts in the following pest risk analysis.

### Pathways for entry

Entry of a pest is the movement of a pest into an area where it is not yet present, or present but not widely distributed and being officially controlled (19). For each pathway, a sequence of events was evaluated: (1) probability of the pest being associated with the individual pathway at origin, (2) probability of survival during transport or storage, (3) probability of surviving existing pest management procedures, and (4) probability of transfer to a suitable host or habitat. Six pathways were identified to assess the likelihood of entry of *P. japonica* into France: (1) import of plants for planting (except seeds, bulbs and tubers) with adhering soil, (2) natural spread, (3) hitchhiking behaviour, (4) import of soil (including potting soil and compost), (5) import of cut flowers and foliage, and (6) import of fruits. The in-depth assessment of each of these pathways of entry was based on data available in the scientific literature, the situation in the invaded countries and the flow of goods towards France (see **Supplementary Material**). We concluded that the likelihood of *P. japonica* entering metropolitan France is high with low uncertainty. The entry will likely occur through natural spread, since the beetle has high flight ability at the adult stage, or by hitchhiking behaviour, given the recent adult sightings in Basel (Switzerland, in 2021) and Baden-Württemberg (Germany, in 2021 and 2022) close to railway track or freight depot (https://gd.eppo.int/reporting/article-7240), in the Valle d’Aosta region (Italy, in 2021) near a motorway service area, and in Sardinia (Italy, in 2021) near the main airport of the island (20). If no regulations were in place, the likelihood of entry would be increased by imports of plants for planting with adherent soil from infested countries, taking into account the probability that the aerial and subterranean stages are associated at the origin and transported, the diversity and volume of the transported goods (such as roses and fruit tree plants especially from Italy), the abilities of the beetles to survive without food (21, 22) during transport, as well as their capacity of transfer to host plants cultivated in France.

### Establishment

**Figure 2:**
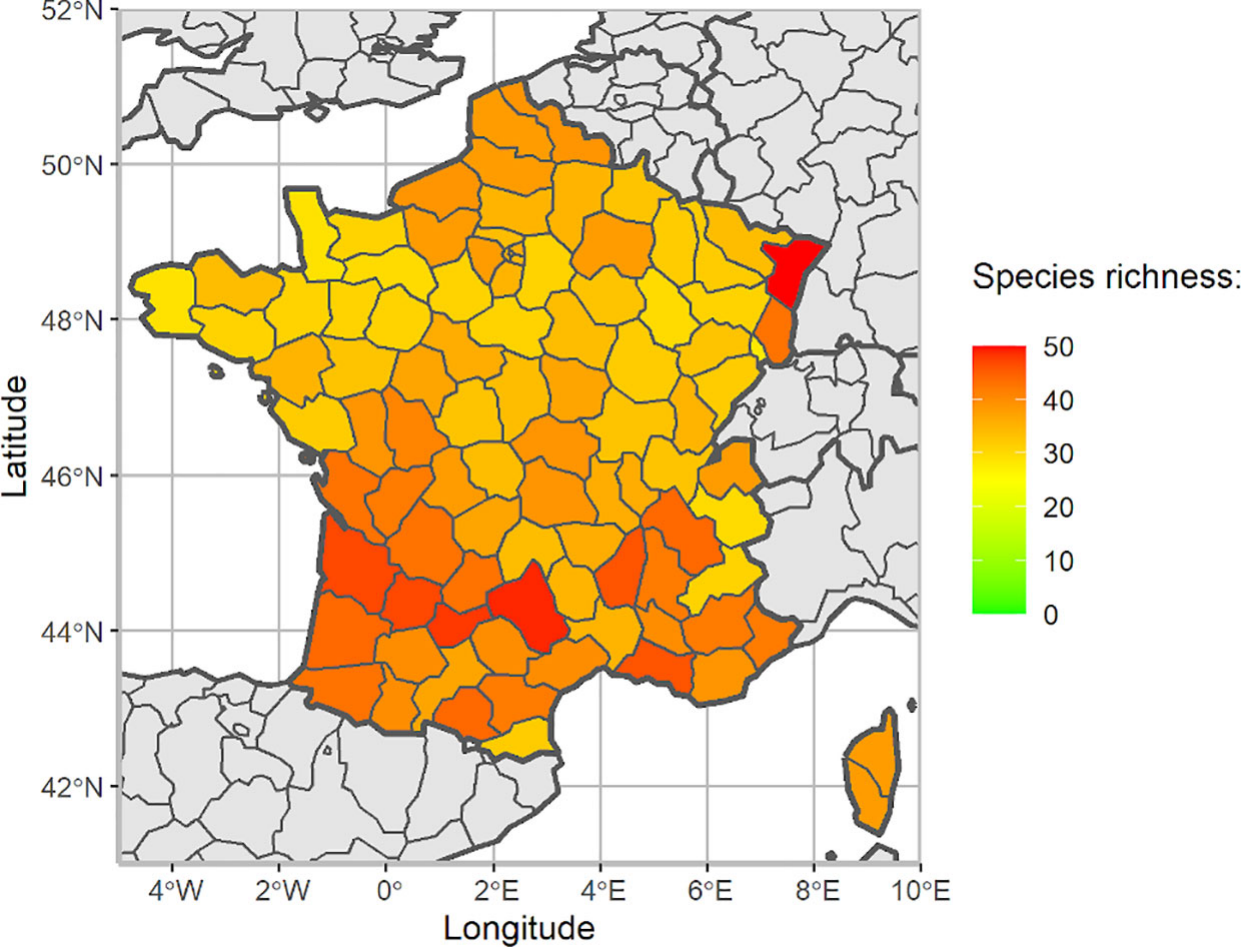
Species richness in main host plants of *P. japonica* in each department (NUTS3 region) of metropolitan France.

Establishment is the perpetuation, for the foreseeable future, of a pest within an area after entry (19). It depends mainly on the presence of host plants and a suitable climate in the PRA area. Four factors that can affect the establishment of *P. japonica* in a new territory have been identified: (1) mild temperature, (2) sufficient humidity for survival and development, (3) presence of host plants and (4) lack of natural enemies. The likelihood of outdoor establishment is considered high, based on the findings from species distribution models (23–26), the high diversity and abundance of host plants in metropolitan France (**Figure 2**), and the limited impact of natural enemies. The uncertainty is considered low. Indeed, the entire French territory, except mountainous areas, is suitable for the establishment of *P. japonica*, because summer rainfall is sufficient, temperature is favourable and many host plants are available. In addition, irrigation practices increase the likelihood of establishment in the less rainy areas of the Mediterranean region. In contrast, the likelihood of establishment in protected conditions (e.g. greenhouses) is considered low with a moderate level of uncertainty. This is due to several points: (1) the facilities concerned are generally small and subject to various pest management methods, (2) there have been no recent reports of *P. japonica* in greenhouses, (3) *P. japonica* populations seem unlikely to be overlooked during regular inspections by growers in indoor conditions.

### Spread

Spread is defined as the expansion of the geographical distribution of a pest within an area (19) and relies on natural spread or human-assisted spread. All previous cases of establishment of *P. japonica* have been followed by spread activity. Natural spread of *P. japonica* is mainly achieved by the flight of adults, with both male and female imagoes having functional wings. However, it appears that pioneer individuals are more likely to be females (27). Data on flight capabilities are essentially based on analyses of the speed of the colonisation front by *P. japonica*, especially in the USA and more recently in Italy. The progression of the invasion front is the result of natural spread, probably coupled with hitchhiking, and suggests spreading capabilities of about 10 km per year in the USA (1, 28, 29). Examining the dynamics of the invasion in Piedmont and Lombardy in Italy (30, 31) provides an equivalent estimate. Human activities, resulting in movements of goods and people, may also favour the long-distance spread of *P. japonica*. In international trade, larvae may be transported in soil attached to the roots of plants for planting, while adult beetles have been intercepted on agricultural products, on packaging and in ships and aircraft (16). Overall, the spread is expected higher than 10 km per year, which is considered a high magnitude according to the EPPO guidance (32). The uncertainty is low, as no barriers to spread within the metropolitan French territory have been identified.

### Impact

Within its current invasion range (data collected from the USA and Italy), the magnitude of impact of *P. japonica* is considered high, with low uncertainty. This results from: (1) high direct damage in terms of loss of yield (fruit crops) and quality (ornamental crops), (2) high indirect costs related to control (especially chemical and biological), (3) the generalist diet of the insect that can affect many production sectors, with significant damage locally. The potential environmental (e.g. increase in phytosanitary treatments, competition with other species) and social impacts (e.g. human health risks, loss of availability of popular ornamental plants) were also considered. In the USA, efforts to control the larval and adult stages were estimated to be around $460 million in 2015 (3). No extensive damage has been recorded in Europe yet, but recent studies (5, 33) suggest substantial potential damage costs. In particular, Straubinger et al. (33) outlined that major grape and wine producing countries like France and Italy would have a potential economic damage of about €92 million and €68 million per year, respectively.

Within the French area at risk of establishment, the magnitude of impact is also considered high with a low uncertainty. This conclusion is mainly supported by (1) the importance of main host plants in terms of area, yield and export volumes, (2) the absence of currently deployed cultural practices that would significantly reduce the impact of *P. japonica* and (3) chemical control is mainly based on a single family of products (pyrethroids). The main point of uncertainty concerns the level of susceptibility of French varieties of the main host plants to *P. japonica*.

Overall, the risk posed by *P. japonica* for the threatened French metropolitan area is considered high with a low uncertainty. This risk is therefore considered as unacceptable and justifies the recommendation of management measures.

## Recommendations for pest risk management

To prevent the entry of *P. japonica* on the French territory, an efficient surveillance strategy is needed to ensure the early detection and to allow the rapid implementation of eradication measures. Using semiochemical-based traps is recommended along the border with infested countries to detect natural spread, as well as near key entry points and transport networks (e.g. national interest markets, airports, air cargo entry points, railways, ports, road hubs, motorways service areas, and unloading areas) to take the risk of hitchhiking behaviour into account. Pheromone traps should be positioned in preferred habitats such as grasslands, vineyards and fruit crops fields located in the close vicinity of entry points. In addition, visual inspections of the aerial parts of the main host plants of *P. japonica* are recommended in these areas along the borders with infested countries. Awareness raising of target stakeholders, including for instance nurserymen and garden owners, is also recommended. The use of pheromone-baited traps is the most reliable monitoring method as it is selective and effective. Compared to visual inspection of aerial parts, trapping ensures permanent coverage of the area to be monitored with a higher level of sensitivity.

In case of first capture, we recommend the deployment of a systematic trapping network with one trap every 1 km on a 10 km square, centred on the trap with the first authenticated capture. This 100-km² area would constitute the reinforced surveillance zone within which an infested zone surrounded by a buffer zone would be delimited. We suggest that the delimitation of the infested zone is continuously adapted according to the locations and amount of new captures, following the concept of weighted barycentre (34). The limits of the buffer zone remain to be determined according to new knowledge on the dispersal capacities of the beetle in the colonised area. We currently suggest a 5-km wide buffer based on the dispersal capacity of *P. japonica* reported in recent studies (35, 36). Since the infested area is a dynamic surface, changing with each discovery of an infestation point, the boundaries of the demarcated area (infested area + buffer zone) would be updated accordingly.

Within the infested area, a combination of measures should be implemented rapidly as part of an eradication strategy: (1) chemical control with the use of authorized active substances against adults and larvae, (2) biological control when available [e.g. entomopathogens such as nematodes (37) or *Paenibacillus popilliae* (3)], (3) cultural practices involving reduced irrigation during the critical oviposition period (38) and tillage in the fall (39, 40). Importantly, many chemical molecules used in the USA to control *P. japonica* are banned in the European Union (notably neonicotinoids), restricting the available arsenal essentially to the family of pyrethroids.

The movement of rooted plants, soil and growing media as well as plant wastes originating from the infested area should be prohibited. The same should apply to plants originating from the buffer zone, which should not be moved out. These actions must be carried out within a short period to increase the chances of eradication. Otherwise, the containment strategy is both time consuming and has, in our opinion, little chance of success, since it would at most slow down the spread of *P. japonica*. Furthermore, the containment strategy involves suppression of *P. japonica* populations within the infested area by chemical control, biological control and mass trapping, whose effectiveness is reduced in case of heavy infestations.

## Discussion and perspectives

Pest risk analyses commissioned at a national level bring out limitations to the precise evaluation of the threat associated with invasive pests and identify opportunities to control their impact. In our study, we addressed specifically the invasive Japanese beetle and its risk to metropolitan France. We assessed the phytosanitary risks in terms of pathways for entry, establishment, spread and economic impacts, and actionable recommendations to mitigate this risk. In this section, we highlight a few points that we believe deserve further attention.

Given the high capacity of *P. japonica* to hitchhike, it is crucial to identify and quantify the movements of goods and people from infested areas to susceptible regions. Eurostat, the statistical office of the European Union, provides extensive information on flows within well-defined pathways, notably those listed in “pathways for entry”. However, specific custom codes do not allow discriminating goods of particular interest regarding the biology and ecology of *P. japonica*, making it difficult to capture only relevant information. Furthermore, whether on a national or European scale, if they exist, the accessibility and visibility of data describing the connectivity of means of transport deserve to be improved. Information on freight by road, rail, air, the road traffic during the period of beetle activity, the list of cars and trucks stop locations, unloading areas, etc., would be valuable to better assess the entry routes of the pest.

The biology and ecology of *P. japonica* have been extensively studied (see for example 1, 41–44). However, further knowledge would be useful, such as the attraction radius of pheromone traps for *P. japonica*. Simple methods exist to estimate this radius (45), useful information to optimise the density of trapping networks. A better understanding of the role of fruit odours on adult feeding attractiveness would also be helpful. Knowing the correlations between the level of susceptibility of host plants and the degree of defoliation should help in assessing the impact on crop yield. It would also be interesting to develop innovative and environmentally sound control strategies that are in line with the European Union commitment to reduce the use of pesticides, such as the use of biological control agents, and the use of attract-and-kill, attract-and-infest, or push-pull strategies.

So far, in continental Europe, *P. japonica* is still confined to a single and relatively small area of about 14000 square kilometres (20) overlapping northern Italy and southern Switzerland. This early stage of the invasion opens up opportunities for successful control, provided that there is greater harmonisation of the surveillance and control strategy on the European level. For example, the insect is rapidly spreading but information on its presence is not fully centralised, making it difficult to develop and share distribution maps. As pointed out by Thompson et al. (46), surveillance strategy and biosecurity measures should be informed by epidemiological processes rather than limited by administrative boundaries. The interception of an adult Japanese beetle in Basel (in 2021), at the intersection of three countries (France, Germany, Switzerland) illustrates these difficulties. In line with recent publications (47–49), we believe that biological invasions could be better regulated by promoting international scientific and technical collaboration to harmonise management practices and regulations.

## Author contributions

This review was conducted by the expert working group “Popillia japonica” of the French Agency for Food, Environmental, and Occupational Health and Safety (ANSES). All authors have contributed equally to the risk assessment presented in this document. SP wrote the first draft and all other authors have provided significant inputs into the text. All authors contributed to the article and approved the submitted version.
