# Supplementary material for: A nationwide pest risk analysis in the context of the ongoing Japanese beetle invasion in Continental Europe: The case of metropolitan France
Source: Front Insect Sci. 2022 Dec 12;2:1079756. doi: 10.3389/finsc.2022.1079756 (PMC10926453; doi:10.3389/finsc.2022.1079756)
Supplement: Supplementary file 1 [file Presentation_1.pptx]

## Slide 1
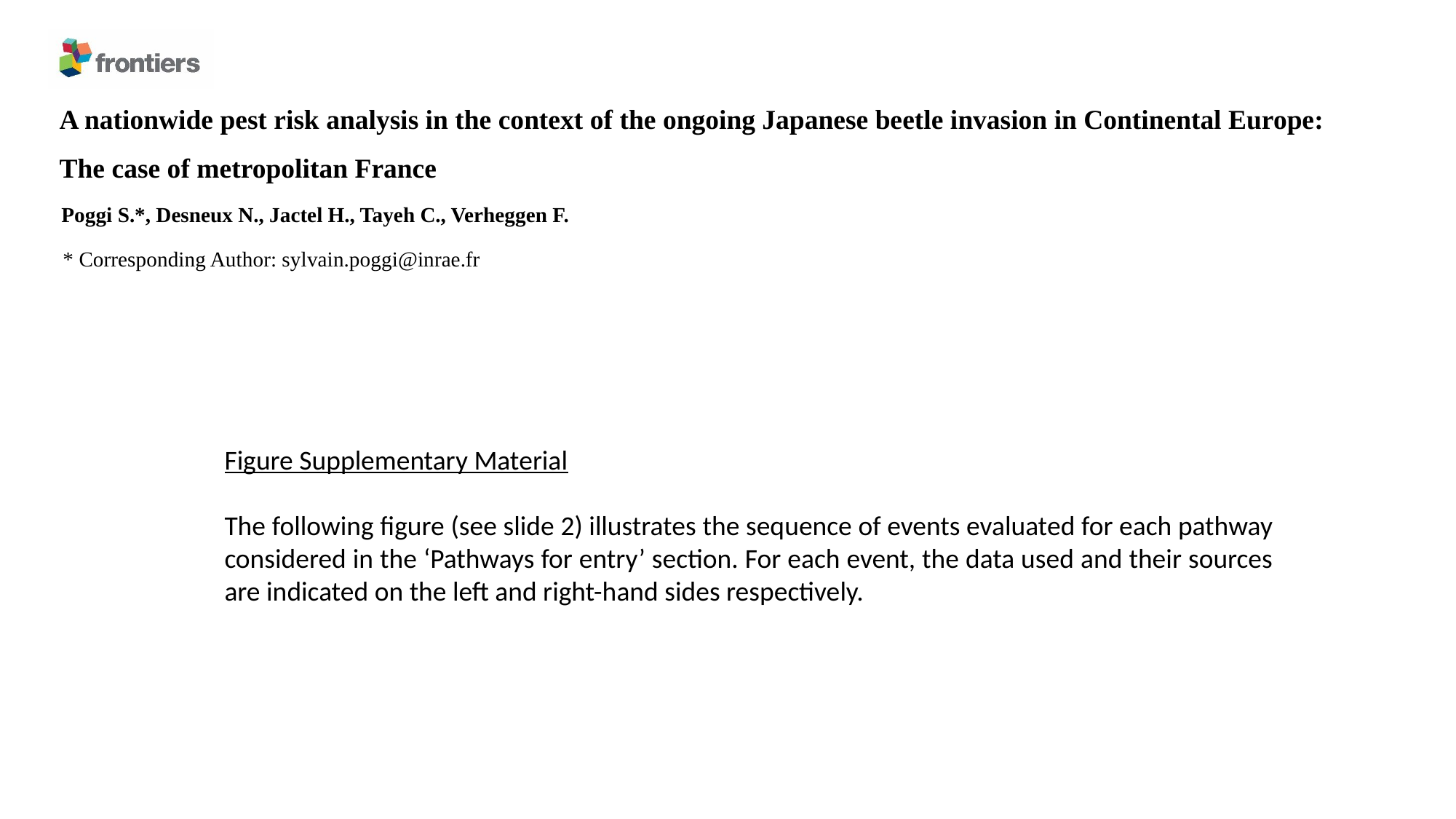

A nationwide pest risk analysis in the context of the ongoing Japanese beetle invasion in Continental Europe: The case of metropolitan France
Poggi S.*, Desneux N., Jactel H., Tayeh C., Verheggen F.
* Corresponding Author: sylvain.poggi@inrae.fr
Figure Supplementary Material
The following figure (see slide 2) illustrates the sequence of events evaluated for each pathway considered in the ‘Pathways for entry’ section. For each event, the data used and their sources are indicated on the left and right-hand sides respectively.

## Slide 2
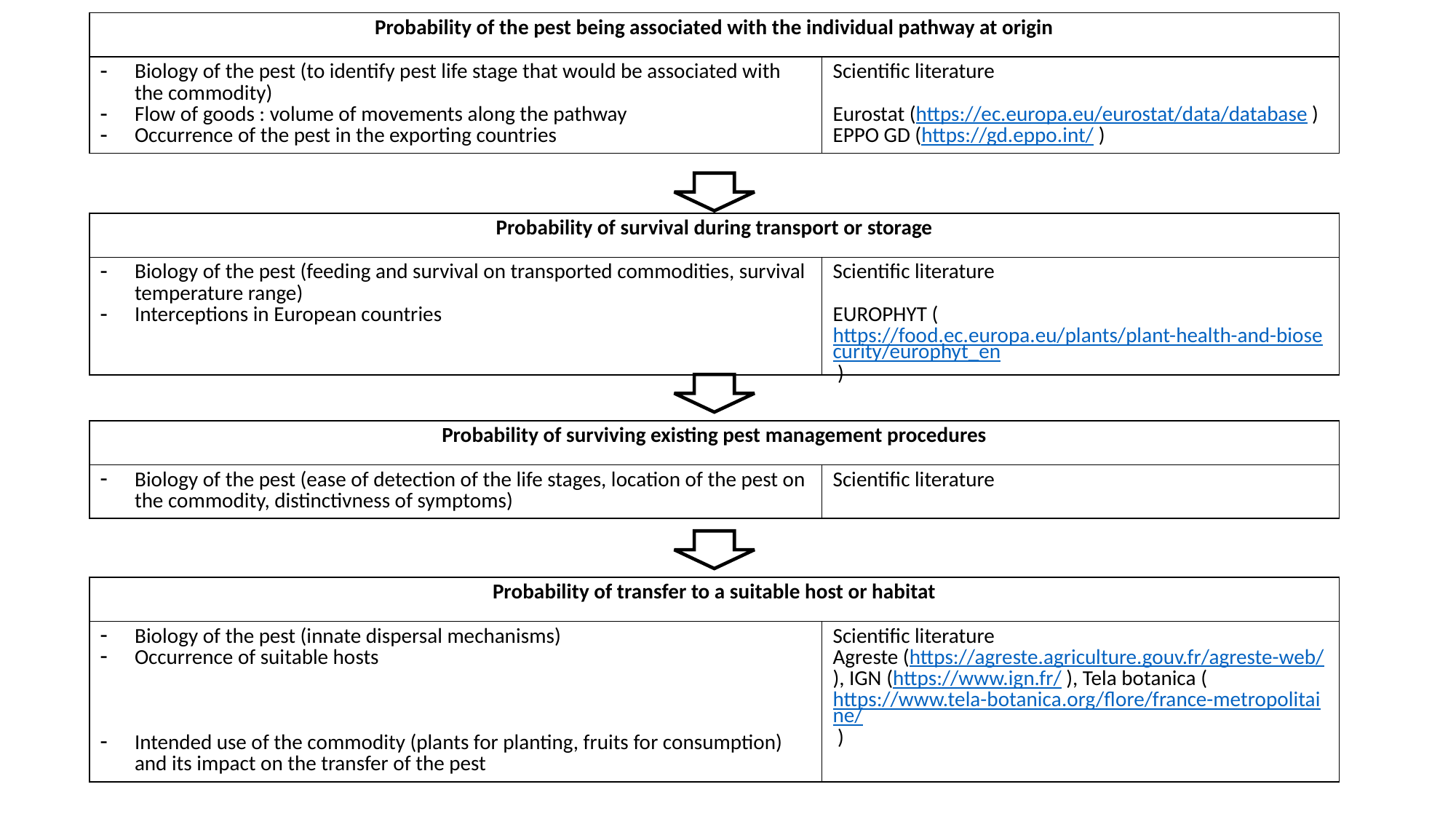

| Probability of the pest being associated with the individual pathway at origin | |
| --- | --- |
| Biology of the pest (to identify pest life stage that would be associated with the commodity) Flow of goods : volume of movements along the pathway Occurrence of the pest in the exporting countries | Scientific literature Eurostat (https://ec.europa.eu/eurostat/data/database ) EPPO GD (https://gd.eppo.int/ ) |
| Probability of survival during transport or storage | |
| --- | --- |
| Biology of the pest (feeding and survival on transported commodities, survival temperature range) Interceptions in European countries | Scientific literature EUROPHYT (https://food.ec.europa.eu/plants/plant-health-and-biosecurity/europhyt\_en ) |
| Probability of surviving existing pest management procedures | |
| --- | --- |
| Biology of the pest (ease of detection of the life stages, location of the pest on the commodity, distinctivness of symptoms) | Scientific literature |
| Probability of transfer to a suitable host or habitat | |
| --- | --- |
| Biology of the pest (innate dispersal mechanisms) Occurrence of suitable hosts Intended use of the commodity (plants for planting, fruits for consumption) and its impact on the transfer of the pest | Scientific literature Agreste (https://agreste.agriculture.gouv.fr/agreste-web/ ), IGN (https://www.ign.fr/ ), Tela botanica (https://www.tela-botanica.org/flore/france-metropolitaine/ ) |
