# Supplementary figures and images for: A nationwide pest risk analysis in the context of the ongoing Japanese beetle invasion in Continental Europe: The case of metropolitan France
Source: Front Insect Sci. 2022 Dec 12;2:1079756. doi: 10.3389/finsc.2022.1079756 (PMC10926453; doi:10.3389/finsc.2022.1079756)

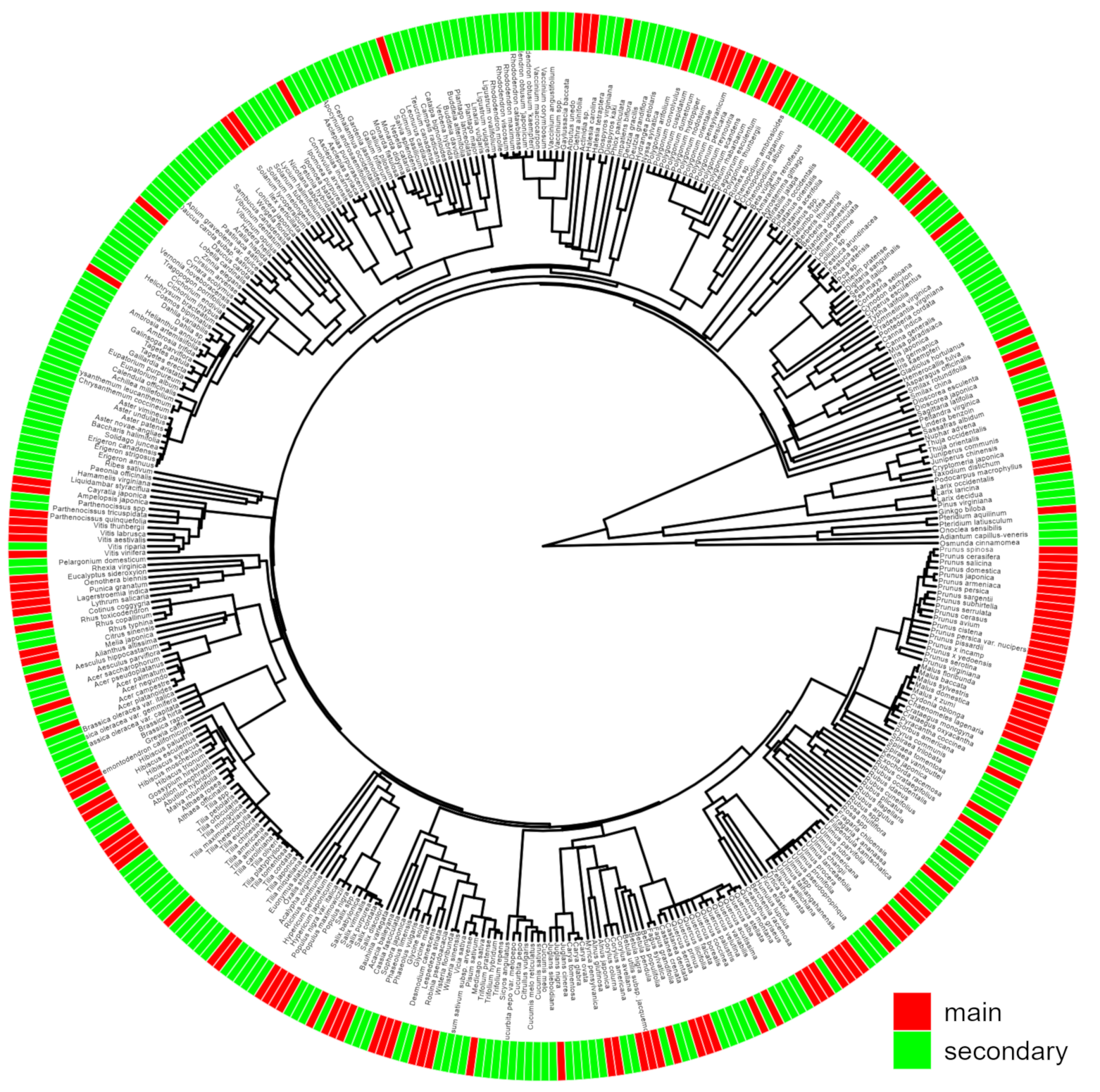

Supplement: Supplementary file 2 [file Image_1.tif]
